# *Allium sativum L.* regulates *in vitro* IL-17 gene expression in human peripheral blood mononuclear cells

**DOI:** 10.1186/s12906-016-1365-9

**Published:** 2016-09-29

**Authors:** Mouna Moutia, Fouad Seghrouchni, Omar Abouelazz, Anass Elouaddari, Abdellah Al Jahid, Abdelhalim Elhou, Sellama Nadifi, Jamal Jamal Eddine, Norddine Habti, Abdallah Badou

**Affiliations:** 1 Laboratory of Hematology and Cellular and Genetic Engineering, Faculty of Medicine and Pharmacy, Hassan II University, Casablanca, Morocco; 2 Laboratory of Experimental Medicine and Biotechnology, Faculty of Medicine and Pharmacy, Hassan II University, Casablanca, Morocco; 3 Laboratory of Cellular Immunology, National Institute of Hygiene, Rabat, Morocco; 4 Cellular and Molecular Pathology Laboratory, Faculty of Medicine and Pharmacy, Hassan II University, 19 Rue Tarik Ibnou Ziad, B.P. 9154 Casablanca, Morocco; 5 Laboratory of Synthesis, Extraction and Physicochemical Study of Organic Molecules, Faculty of Sciences Ain Chock, Hassan II University, Casablanca, Morocco; 6 Research Team Health and Environment, Cadi Ayyad University, Polydisciplinary Faculty, Safi, Morocco

**Keywords:** *Allium sativum*, Peripheral blood mononuclear cells, Anti-inflammation, Cytokines, Gene expression, Cell proliferation, Cell toxicity

## Abstract

**Background:**

*Allium sativum L.* (A.S.) “garlic”, one of the most interesting medicinal plants, has been suggested to contain compounds that could be beneficial in numerous pathological situations including cancer. In this work, we aimed to assess the immunomodulatory effect of A.S. preparation on human peripheral blood mononuclear cells from healthy individuals.

**Methods:**

Nontoxic doses of A.S. were identified using MTT assay. Effects on CD4+ or CD8+ T lymphocyte proliferation were studied using flow cytometry. The effect of A.S. on cytokine gene expression was studied using qRT-PCR. Finally, qualitative analysis of A.S. was performed by HPLC approach. Data were analyzed statistically by one-way ANOVA test.

**Results:**

The nontoxic doses of A.S. preparation did not affect neither spontaneous nor TCR-mediated CD4+ or CD8+ T lymphocyte proliferation. Interestingly, A.S*.* exhibited a statistically significant regulation of IL-17 gene expression, a cytokine involved in several inflammatory and autoimmune diseases. In contrast, the expression of IL-4, an anti-inflammatory cytokine, was unaffected. Qualitative analysis of A.S. ethanol preparation indicated the presence of three polyphenol bioactive compounds, which are catechin, vanillic acid and ferulic acid.

**Conclusion:**

The specific inhibition of the pro-inflammatory cytokine, IL-17 without affecting cell proliferation in human PBMCs by the *Allium sativum L.* preparation suggests a potential valuable effect of the compounds present in this plant for the treatment of inflammatory diseases and cancer, where IL-17 is highly expressed. The individual contribution of these three compounds to this global effect will be assessed.

**Electronic supplementary material:**

The online version of this article (doi:10.1186/s12906-016-1365-9) contains supplementary material, which is available to authorized users.

## Background

T cells represent about 70 % of human Peripheral Blood Mononuclear Cells (PBMCs), in addition to monocytes, natural killer “NK” cells and B cells. Among T lymphocytes, CD4+ T cells are subdivided into various subpopulations such as Th1, Th2 and Th17 cells. Each of these subpopulations has its own set of immunological functions, allowed by a specific group of produced cytokines. For instance, Th1 cells are in charge of intracellular pathogen eradication [1, 2]; but are also involved in the progress of autoimmune diseases and chronic inflammatory disorders [3]. They are characterized by high secretion of IFN-γ and IL-2. Th2 cells are implicated in humoral immunity and provide protection against parasites. They are engaged in human allergic inflammation; its initiation, maintenance and amplification [4] via the production of IL-4, IL-10 and IL-13 [4, 5]. Th17 are involved in the clearance of extracellular bacteria and fungi, due to their capacity to recruit and activate Neutrophils [6]. They are known for their production of IL-17A and IL-17 F. However, Th17 cells are also implicated in promoting the pathogenesis of cancer [7], several autoimmune and inflammatory diseases [8]; such as rheumatoid arthritis, multiple sclerosis, inflammatory bowel disease, psoriasis and contact dermatitis [9, 10]. Our main interest is to identify phyto-molecules capable of regulating specific pro-inflammatory cytokines such as IL-17, which is implicated in various pathological situations including autoimmunity [8] and cancer [7, 9, 10].

*Allium sativum L.*, a member of the Liliaceae family, is used universally as a flavoring agent in traditional medicine and to enhance physical and mental health [11–15]. Some reports also showed that a protein fraction of aged A.S. extract enhanced NK cell activity and cytotoxicity of macrophages towards tumor cells [16]. Another study demonstrated that ingestion of A.S. oil modulated the production of T helper cytokines in rat cervical lymph nodes [17]. Accordingly, A.S. and its specific ingredients, organosulphur compounds, are thought to affect the immune system [18].

In this paper, we aimed to assess the immunomodulatory effect of A.S. extract on human peripheral blood mononuclear cells. Doses of A.S., which did not cause cytotoxicty and did not affect cell proliferation, exhibited a marked regulation of the expression of the pro-inflammatory cytokine IL-17, known to be involved in numerous autoimmune and inflammatory diseases.

## Methods

### *A.S.* extract preparation

*Allium sativum L*. “garlic” bulbs were harvested from a botanical garden in the Faculty of Medicine and Pharmacy of Casablanca, Morocco. The plant was identified by a professor of Botany affiliated to the Biology department of the Polydisciplinary Faculty of Safi. A voucher of the plant specimen has been deposited in the herbarium of the department of Botany of the Scientific Institute of Rabat, Morocco under the reference number 97997. One-hundred grams of peeled dry garlic bulbs were mashed and incubated in ethanol (Sigma, USA) for 24 h (twice) at room temperature. Extracts were then concentrated by rotary. The obtained ethanolic extracts were stored at -20C° until use.

### Analysis of polyphenols by HPLC

Qualitative analysis of standard phenolic compounds in A.S. ethanolic extract was performed using high performance liquid chromatography (HPLC) type JASCO PU-1580, equipped with a UV detector / Vis type JASCO 875 UV and a data treatment station Azur version 3.0.3.0. The extract was evaporated to dryness at 40 °C and then taken up with a mixture of distilled water - acetonitril (88–12 %). After vigorous stirring, mixtures were filtered by passing the solution through Whattman nylon membrane. Analysis conditions used were: Column: C18 (1.7 μm 2,1 × 150 mm), UV Wavelength: 285 nm, 1 ml / min, gradient elution program was set as water /acetonitrile (88–12 %). The solution of the polyphenol mixture: Catechin, rutin, vanillin, vanillic acid, caffeic acid, syringic acid and ferulic acid was prepared by dissolving 1–5 mg of each polyphenol in 1 ml of the acetonitril - water 12–88 %. The final solution was filtered through a Whattman nylon membrane 0.22 μm.

### PBMC preparation and cell culture

Peripheral blood mononuclear cells “PBMCs” were separated from fresh venous blood collected from healthy donors as previously described [19]. PBMCs isolation was performed by centrifugation on Ficoll- histopaque 1077 (Sigma, USA), at 900 g for 25 min at 18–20 °C, then washed three times by centrifugation, and suspended in RPMI 1640 media (sigma, USA) with L-glutamine supplemented with 10 % heat inactivated newborn calf serum (sigma, USA), 26.3 g/l penicillin and 4.2 g/l streptomycin. Cells were counted using a hemocytometer and standard Trypan blue exclusion method and observed under microscopy. Cells were harvested in plat culture at 37 °C in a humid atmosphere containing 5 % CO_2_ (Heracell 150i incubator CO_2_, Thermosciences, France) with different doses of A.S. preparation, with or without 5 μg/ml of phytohaemagglutinin (PHA; Sigma, USA) or plat bound monoclonal anti-CD3 (Okt3, 0.1 μg/ml).

### MTT assay

Cytotoxicity of A.S. extract to PBMCs was evaluated by MTT (3-(4, 5 dimethylthiazol-2-yl)-2, 5 diphenyltetrazolium bromide) assay [19, 20]. The test was performed in 96-well plates (Hiwaka, Japan). Triplicate cultures in 200 μl of cell suspension (10^5^cells/well) were incubated in the presence of different doses of A.S. extract with or without stimulation using PHA or anti-human CD3 Okt3 monoclonal Ab. After 4 days of culture, 20 μl of MTT solution (5 mg/ml, Sigma, USA) was added to the wells and incubated for additional 4 h under the same conditions. After removing the plates and centrifuging them at 800 × g for 20 min, supernatant was removed and blue formazan crystals were solubilized by adding 100 μl of DMSO (Sigma, USA) under agitation. Optical Density of cells with A.S. extract was compared to that of control cells. Low optical density readings reflected low coloration intensity, due to the inability of non-viable cells to metabolize MTT salts.

### Proliferation assay

PBMCs (10^6^cell/ml) were harvested in 96-well plates. A.S. extract was used at final concentrations of 1 and 2 μg/ml, with or without PHA and with or without anti-CD3 (Okt-3). Cells were labeled before culture with 5, 6-carboxyfluorescein diacetate succinimidyl ester (CFSE) (Sigma, USA) at a concentration of 5 *μ*M, according to a previous paper [21]. Afterward, labeled cells were washed twice with RPMI containing 5 % heat inactivated newborn calf serum. After 4 days of culture, PBMCs were labeled with Anti-CD4 (PrCP, BDBiosciences, France) and anti-CD8 (APC BD Biosciences, France), in order to evaluate proliferation of CD4+ and CD8+ lymphocyte subsets. Analyses were performed using FACS (BD FACSCalibur Flow Cytometer, BD Biosciences, France) and FlowJo software (Ashland, USA). One hundred thousand events were analyzed per sample.

### Gene expression quantification by RT-qPCR

#### Total RNA isolation

TRIzol (Sigma, France) was used to isolate total ARN from cells cultured for 18 h with different doses of A.S. extract in the presence or absence of PHA. Isolated RNA was solubilized in DEPC Treated Water (Invitrogene, France) and measured with spectrophotometer (NanoVue™ Plus Spectrophotometer GE Healthcare UK Limited, UK).

### Reverse Transcription (RT)

0.5 μg of total ARN was used to synthesize cDNA using M-MLV reverse transcriptase (Reverse Transcriptase Super Scripte III, 10000 units, Invitrogene, France) in a 20 μl reaction mixture according to the manufacturer’s instructions. 1 μl of oligo dT_20_ (50 μM) and 1 μl of dNTP (10 mM of each) were added and the mixture was incubated at 65 °C for 5 min to break the secondary structure of RNA; and then put on ice for 5 min. 4 μl of 5X Reverse Transcriptase buffer, 1 μl de DTT (100 mM), 1 μl of RNase Inhibitor (RNasin OUT 5000 units, Invitrogene, France) and 1 μl of M-MLV reverse transcriptase were subsequently added and the mixture was incubated at 50 °C for 60 min, then 70 °C for 15 min.

### Real-time PCR assays

Amplifications were performed using Syber® Green PCR Master mix (Applied Biosystems™, life Technologies, France) as recommended by the manufacturer’s manual. Using primers at 500nM for all genes, PCR was programmed as follows: 10 min at 95 °C for polymerase activation and sample denaturation; then 40 cycles of 15 s at 95 °C and 60 s at 60 °C for annealing and extension. Relative quantification of gene expression was analyzed by real-time PCR using real time Fast 7500 (Applied Biosystems™). β-actin was used as internal control. Primers used are as follows; β-actin: forward GAGATGGCCACGGCTGCTT, reverse GCCACAGGACTCCATGCCCA (transcript size: 446 bp), IL-17: forward ATCTCCACCGCAATGAGGAC, reverse: GTGGACAATCGGGGTGACAC (transcript size: 77 bp), IL-4: forward ATGGGTCTCACCTCCCAACTGCT, reverse GTTTTCCAACGTACTCTGGTTGGC (transcripts size: 506, 357, 405 bp) and IFN-γ forward GAAGAATTGGAAAGAGGA, reverse CGACAGTTCAGCCATCAC (transcript size: 265 bp). Fluorescence readings at the end of the extension phase of each cycle were used to estimate the values for the threshold cycles (Ct). The Ct values for each gene were converted into relative quantification (2^-ΔΔCt^) using the machine 7500 v2.0.6 software. Samples of untreated and unstimulated cells were used as a calibrator for unstimulated treated cells samples. On the other hand, untreated stimulated samples were used as a calibrator for stimulated and treated cell samples. PCR negative control (with no template) was included for each pair of primers.

### Statistical analysis

All data were expressed as means +/− standard deviations. Groups were compared using one-way ANOVA followed by Bonferroni’s Multiple Comparison test, with a level of significance set at *p* < 0.05. GraphPad Prism 5.0 software was used for all statistical analyses.

## Results

### Evaluation of the cytotoxicity of *Allium sativum* preparation to human PBMCs

**Fig. 1:**
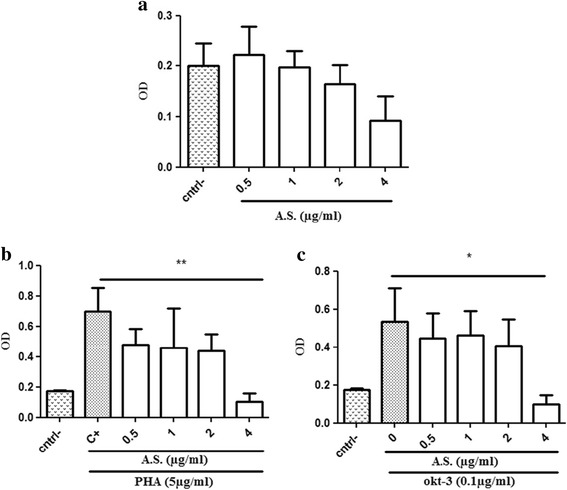
Assessment of *Allium sativum* preparation cytotoxicity on human PBMCs. Human PBMCs were cultured for 4 days in the absence (cntrl-) or the presence of different doses of *Allium sativum* extract (**a**). In another series of experiments, PBMCs were left untreated or were treated with A.S. extract in the presence of PHA (**b**) or Okt-3 mAb (**c**). 4 days later, MTT assay was performed to evaluate cytotoxicity. Experiments shown in **a**, **b** and **c** are representative of five, five and three independent experiments respectively. Data were analyzed using one way ANOVA test. (*, **) indicate *P* values of less than 0.05 and 0.01 respectively

In order to assess the immunomodulatory effect of the A.S. preparation, we started by evaluating cell toxicity of different doses of A.S.. Human PBMCs were treated with increasing doses of A.S. extract in the presence or the absence of stimulation (PHA or anti-CD3 mAb). Figure 1a shows that A.S. extract was not toxic to PBMCs at doses ranging between 0.5 and 4 μg/ml. On the other hand, A.S. extract was found not toxic to PBMCs stimulated with PHA up to certain doses (0.5 to 2 μg/ml). However, 4 μg/ml exhibited a significant toxicity to cells as shown in Fig. 1b. Similar pattern was observed with cells stimulated with anti-CD3 mAb (Fig. 1c). As a result, we concluded 4 μg/ml as a significantly toxic dose of A.S. extract to stimulated human PBMCs.

### Effect of *Allium sativum* preparation on PBMC Proliferation

**Fig. 2:**
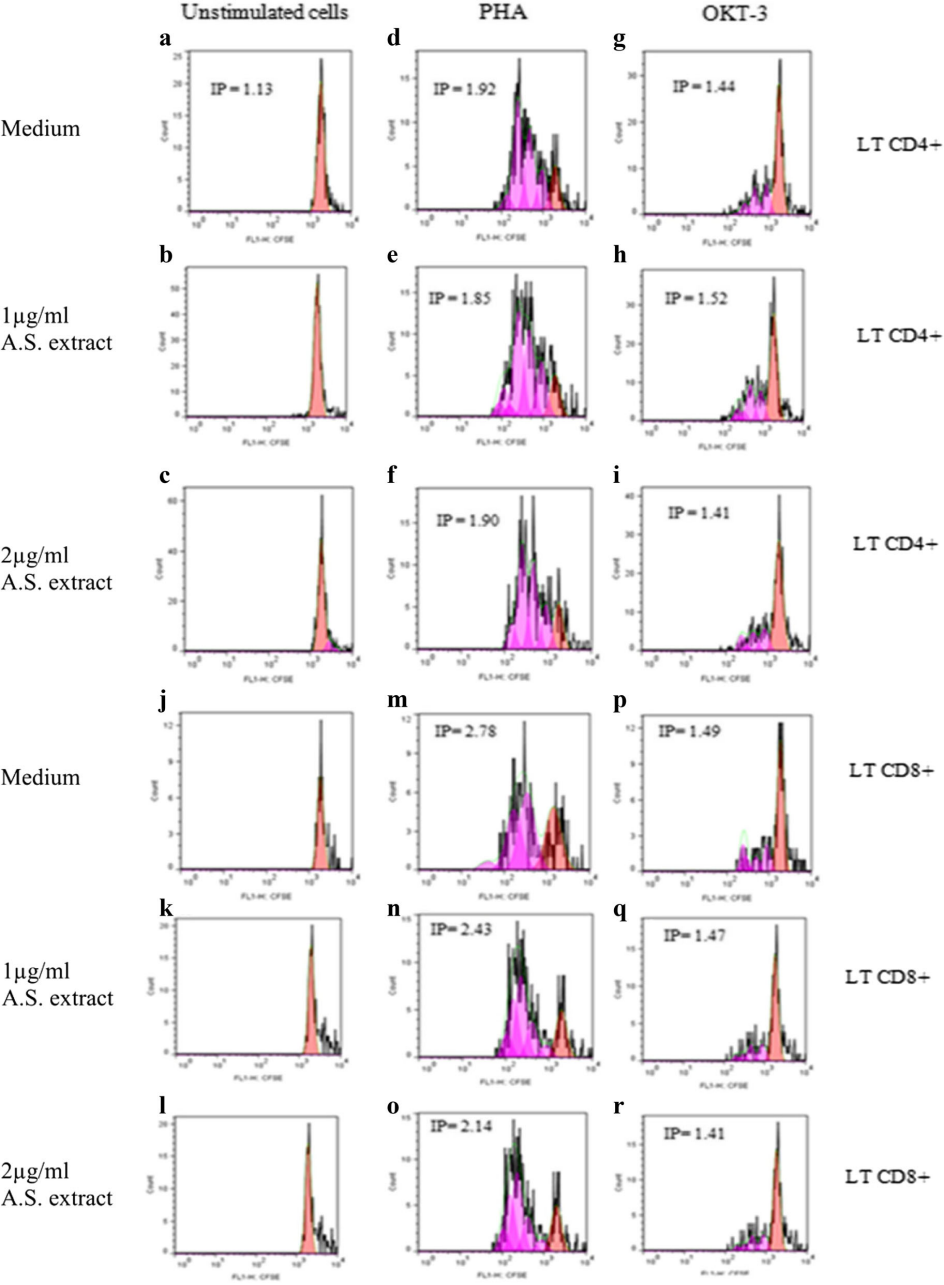
Effect of *Allium sativum* extract on human PBMCs proliferation. Panels from **a** to **i** show an example of data generated by CFSE and anti-CD4 stained PBMCs. Panels from **j** to **r** show an example of data generated by CFSE and anti-CD8 stained PBMCs. Cells were cultured in different conditions of stimulation and treated with two doses of *Allium sativum* (A.S.) extract for 4 days. **a** and **j** untreated PBMCs **b** and **k**, **c** and **l** PBMCs treated with A.S. extract at 1 and 2 μg/ml respectively. **d** and **m** PBMCs stimulated with PHA. **e** and **n**, **f** and **o** cells were stimulated with PHA and treated with A.S. extract at 0.5 and 1 μg/ml respectively. **g** and **p** cells were stimulated with Okt-3 mAb at 0.1 μg/ml. **h** and **q**, **i** and **r** PBMCs stimulated with Okt-3 mAb and treated with A.S. extract at 1 and 2 μg/ml respectively. Each peak represents a cycle of cell division. The curves generated by the CFSE profile were analyzed using the proliferation platform of the FlowJo software. Data shown represent results of 4 independent experiments. IP indicates index of proliferation calculated with FlowJo software

Subsequently, we tested the effect of the non toxic doses of A.S. extract on human PBMC proliferation by using CFSE combined to flow cytometry. To test whether A.S. is able to induce PBMC (more specifically CD4+ and CD8+ T lymphocytes) proliferation in the absence of mitogenic stimulation, unstimulated CFSE-labeled PBMCs were incubated with A.S. preparations at two doses (1 and 2 μg/ml) for 96 h. Our results showed that A.S. did not affect significantly T lymphocyte proliferation at the non toxic doses tested (Fig. 2 a, b, c, j, k, l; and Additional file 1: Figure S1a and c). In order to test whether A.S. is able to inhibit or enhance PHA or anti-CD3 –stimulated CD4+ and CD8+ T cells proliferation, CFSE-labeled PBMCs were stimulated by PHA or anti-CD3 mAb (Okt-3), in the absence or presence of A.S. extract (Fig. 2 d, e, f, m, n, o, g, h, i, p, q, r; and Additional file 1: Figure S1 a, b, c et d). These data showed that A.S. did not affect significantly CD4+ or CD8+ T lymphocyte proliferation.

### Significant regulation of IL-17 gene expression by *Allium sativum*

**Fig. 3:**
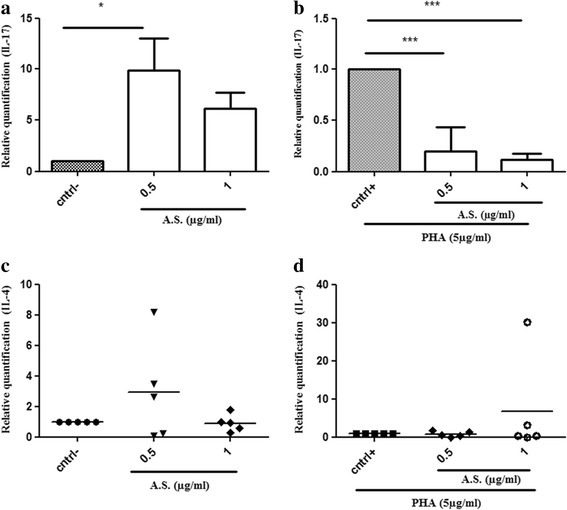
Effects of *Allium sativum* extract on IL-17 and IL-4 gene expression in PBMCs. IL-17 (**a**) and IL-4 (**c**) transcripts were evaluated in PBMCs treated with two doses of A.S. IL-17 (**b**) and IL-4 (**d**) transcripts were assessed in PBMCs stimulated with PHA in the absence or presence of two doses of A.S.. Data were represented in mean ± S.D. Data were analyzed using the one-way ANOVA test. (*, ***) indicate *P* values of less than 0.05 and 0.001 respectively. Data shown in a, b, c and d are representative of four, three, five and five independent experiments respectively

To assess the effect of *Allium sativum* on pro- and anti-inflammatory cytokine gene expression, we treated PBMCs from healthy donors, for 18 h, with non toxic doses of A.S. preparation with or without PHA stimulation. Results in Fig. 3a indicate that a single treatment with A.S. extract stimulated significantly the expression of the pro-inflammatory cytokine, IL-17. We could detect an increased expression of IL-17 gene in 4 out of 6 donors tested. In all these 4 donors, we have observed an increase in IL-17 transcripts upon treatment with A.S.. Interestingly, when PBMCs were stimulated with PHA, treatment with A.S. showed a significant and dose dependent decrease in IL-17 gene expression (Fig. 3b). On the other hand, we could not detect any statistically significant effect of A.S. on IL-4 gene expression (known to be an anti-inflammatory cytokine), either in the absence (Fig. 3c) or presence (Fig. 3d) of a T lymphocyte activator such as PHA. It is to note that a trend of increase in IL-4 gene expression was observed in some donors both in the absence (Fig. 3c) and the presence (Fig. 3d) of PHA, but this effect was not significant. We also tempted to quantify the expression of IFN-γ gene in our experiments, but we could not detect its expression in our tested donors (data not shown); and thus no conclusions could be drawn for this cytokine. Altogether, these data suggested that A.S. preparation exhibited a significant regulation of IL-17 gene expression and that this effect would depend on the activation state of T lymphocytes.

### Analysis of polyphenols and flavonoids in *Allium sativum* extract by HPLC

**Fig. 4:**
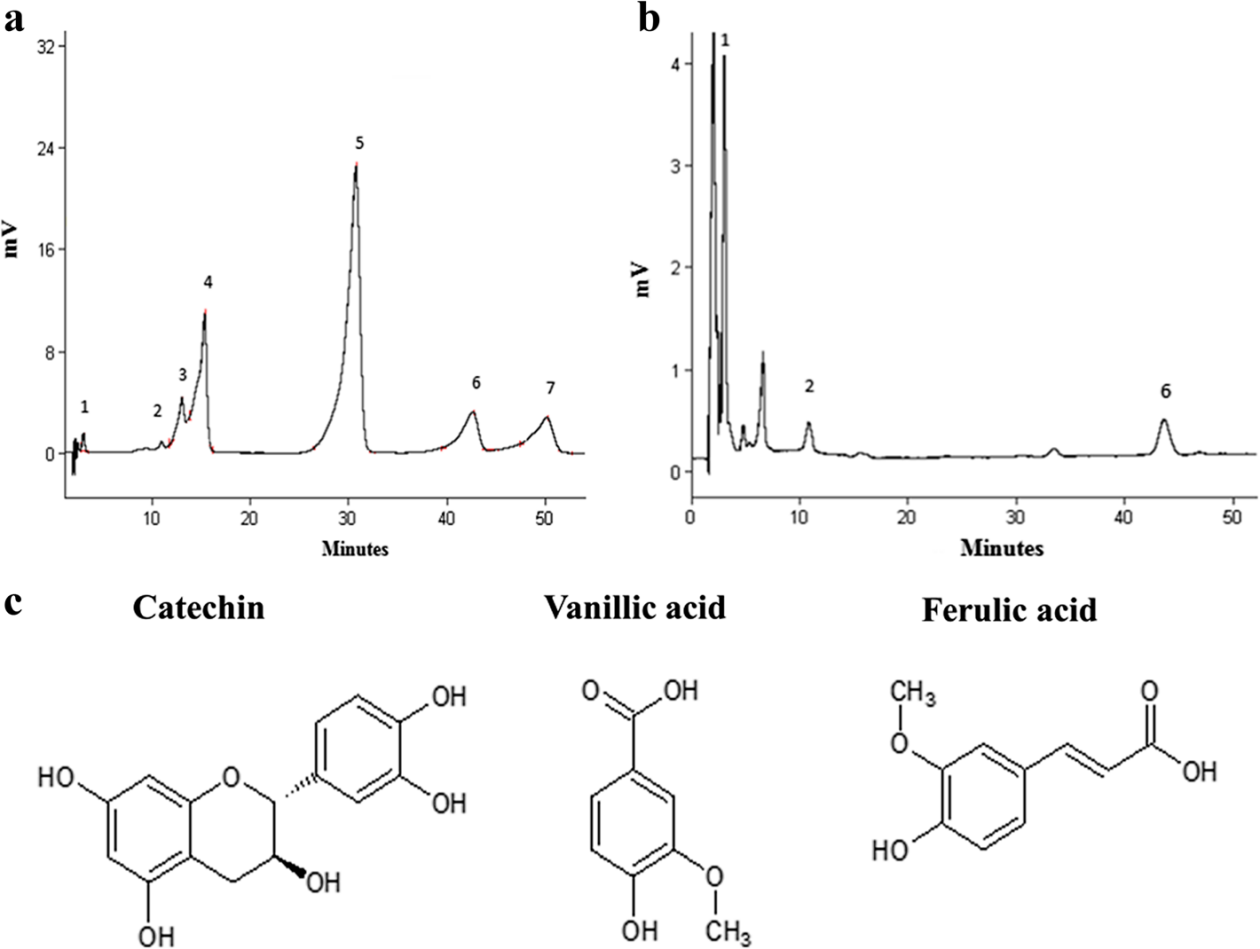
**a** Chromatogram of 7 available polyphenol and flavonoid standards monitored at 280 nm and identified by retention time (minutes) 1: catechin, 2: vanillic acid, 3: cafeic acid, 4: syringic acid, 5: rutin, 6: ferulic acid, 7: vanillin. **b** HPLC chromatogram of A.S. extract obtained under optimum conditions: 88 % water and 12 % acetonitril, 50 min. Peak n^o^. 1: catechin, 2: vanillic acid and 6: ferulic acid were identified. **c** The chemical structure of the three identified compounds

With the aim of identifying the bioactive compounds of *Allium sativum* extract responsible for the observed effect, A.S. ethanolic preparation was analyzed by HPLC at 285 nm, to determine the presence of polyphenols and flavonoids. A mixture of seven standards was used in this experiment. Interestingly, three compounds, catechin, vanillic acid and ferulic acid, were identified from the extract by matching their retention time against those of the standards. Peak assignment was confirmed by injection of standards. Figure 4a shows the HPLC chromatogram of a mixture of 7 polyphenol standards for peak comparison with the chromatogram of the A.S. extract (Fig. 4b). The HPLC chromatogram of A.S extract in Fig. 4b displayed 7 peaks detected at 285 nm. Peaks 1, 2 and 6 were identified as catechin, vanillic acid and ferulic acid, respectively. The chemical structure of each of the identified compounds is elucidated in Fig. 4c.

To summarize, this study showed that A.S. extract contains compounds, which could be used in non toxic doses and without affecting T cell proliferation, to modulate IL-17 gene expression by significantly stimulating its expression in the absence of prior PBMC activation; and to significantly inhibit its expression when PBMCs are stimulated with a mitogen such as PHA. Interestingly, in the same experimental conditions, A.S. does not affect the expression of the anti-inflammatory cytokine, IL-4. Furthermore, these data showed that our A.S. extract contains 3 polyphenol compounds, catechin, vanillic acid and ferulic acid, which could be responsible for this potentially interesting effect.

## Discussion

For many years, A.S. has been acknowledged as an important folk medicine with a favorable effect against a large number of pathologic conditions. In this work, we aimed to evaluate the potential in vitro immunomodulatory effect of A.S., on PBMCs from healthy donors. We started by defining a range of non toxic doses using the MTT assay. These doses were used to evaluate cell proliferation and to quantify pro and anti-inflammatory cytokine gene expression. Our data showed that doses from 0.5 to 2 μg/ml of A.S. ethanolic extract were not toxic to cells under any of the used conditions. The same doses were used to test whether our A.S. extract exhibited any effect on PBMCs proliferation. Our results showed that A.S. extract did not affect neither human CD4+ nor CD8+ T cell proliferation induced by PHA, as shown by flow cytometry, which is consistent with data reported by others [16] when cells were stimulated by PHA. While in the same time, other data showed divergent results. Since Morioka et al. showed that A.S. extract enhanced ConA-induced proliferation of human T lymphocytes [16]. Other data revealed that several organosulfur compounds extracted from A.S. displayed a cell proliferation modulation, such as ajoene that exerted a strong inhibitory effect on PHA, phorbol myristate acetate and anti-CD3 mAb -induced proliferation of human lymphocytes [22, 23]. Colic et al. also demonstrated that a protein fraction from A.S. aqueous extract exhibited an enhancement of T-cell proliferation in mouse splenocyte cultures stimulated with PHA. As further explained in [24, 25], A.S. extracts and their compounds had a dual role on mice and rat T lymphocyte proliferation. In fact, higher concentrations (50 μg/ml) inhibited T cell proliferation triggered by ConA and at lower concentrations (3–12.5 μg/ml) of the extract, amplified the ConA-induced proliferative response of T cells [25]. It was also well demonstrated that aged A.S. extract enhanced the proliferation of spleen cells. Aged A.S. extract slightly improved ConA- induced proliferation of mice spleen cells, but did not increase the effects of the other mitogens, pokeweed mitogen (PWM) and lipopolysaccharides (LPS) [26]. This divergence might depend on different stimulation types, on the cell type used, and the A.S. preparation method used during these experiments.

As for cytokine gene expression, we could not detect any significant effect on IL-4 gene expression. While previous studies reported an increase in IFN-γ and IL-4 production in lymphocytes from rat spleens upon oral A.S. consumption [27]; and an increase in IL-4 when lymphocytes from rat spleens were stimulated by phorbol myristate acetate and treated with aqueous and ethanol preparations made from garlic powder [28]. A.S. powder extracts were found to reduce the level of pro-inflammatory cytokines, IL-1β and TNFα, in human blood sample supernatants, while the expression of the anti-inflammatory cytokine IL-10 was unchanged [29].

Our present study is the first report showing the ability of A.S. compounds to inhibit IL-17 gene expression. The present data showed an inhibition of the pro-inflammatory cytokine, IL-17 gene expression without affecting the anti-inflammatory cytokine IL-4, which might suggest a specific potential regulation of IL-17 that could be useful in controlling several inflammatory and autoimmune diseases, such as multiple sclerosis, rheumatoid arthritis and cancer. For example, it was shown that IL-17 plays a pivotal role in the development of central nervous system inflammation in multiple sclerosis, where it was found to correlate with active lesions [30] and to be up-regulated in chronic lesions in multiple sclerosis patients [31, 32]. IL-17 was also shown to be involved in the pathogenesis of rheumatoid arthritis [33]. Indeed, a high level of IL-17 was found in synovium and synovial cultures from rheumatoid arthritis patients [34, 35]. IL-17 appeared to activate and enhance all mechanisms of tissue injury and to up-regulate and/or synergize with local inflammatory mediators such as IL-6 [36, 37], IL-1β and TNF-α [37, 38]. Furthermore, there are suggestions that in pulmonary asthma there are higher levels of intracellular IL-17 and increased numbers of IL-17-producing cells (T lymphocytes and eosinophils in broncheoalveolar lavage) relative to healthy controls both locally and within the circulation [39]. Higher amounts of IL-17 has also been revealed in many different types of human tumors including lymphoma, melanoma, breast, colon, gastric, hepatocellular, pancreatic, ovarian and prostate cancers. Overall, the presence of Th17 cells and/or IL-17 has been reported to be a poor prognostic indicator [40]. Similar observations have also been made in different mouse models of cancer [40]. Wilke et al. explained how IL-17 has become an interesting therapeutic target in many autoimmune diseases [41].

Because there is an ongoing debate concerning whether organosulfur compounds or other constituents such as polyphenols and flavonoids, mediate the protective effect attributed to garlic [29], we carried out a qualitative analysis of the A.S. extract by HPLC. This showed the presence of two phenolic acids “vanillic acid and ferulic acid” and a flavonoid of the flavonols class which is “catechin”. These phenolic compounds are known to have interesting immunomodulatory effects but none of them was tested on the expression of IL-17 from human PBMC. Vanillic acid, which is a benzoic acid derivative and an oxidized form of vanillin formed during the transformation of vanillin to ferulic acid [42–44], has been proved to enhance the activity of lymphocyte proliferation and IFN-γ secretion in human PBMCs [45] and to exhibit a hepatoprotective effect by its suppressive action on immune-mediated liver inflammation in ConA-induced liver injury in mouse [44, 46]. As for ferulic acid, it was found to have a chemopreventive activity against coronary heart disease, thrombosis, mutagenesis and carcinogenesis, in addition to an anti-cancer activity against colon and rectal cancer [47]. Catechin is a major compound of flavonoids, which are known for their ability to control lymphocyte proliferation and to modulate IL-2 and IFN-γ secretion in human PBMCs and mouse splenocytes [48]. It was also found that catechin ameliorates cardiac dysfunction, in a rat model of chronic heart failure, by regulating the unbalanced level of IL-17/IL-10 [49].

## Conclusion

*Allium sativum L.* preparation exhibiting a specific inhibition of the pro-inflammatory cytokine IL-17, without affecting cell proliferation in human PBMCs indicates a potential valuable effect of the compounds present in this plant to treat inflammatory diseases and cancer, where IL-17 is highly involved. The individual contribution of these identified compounds to this interesting global inhibitory effect is under investigation and could unravel a pivotal therapeutic role.

## Additional files

Additional file 1: Figure S1. Effect of *Allium sativum* extract on human PBMC proliferation. CD4 T cell division was evaluated by CFSE staining and flow cytometry when PBMCs were treated with or without A.S. at two different doses in the absence or presence of PHA (a) or Okt-3 mAb (b). CD8 T cell division assessed by CFSE staining and flux cytometry within PBMCs treated with or without A.S. at two doses in the absence or presence of PHA (c) or Okt-3 mAb (d). Data shown are representative of 4 independent experiments. Percentage of cell division was calculated with FlowJo software. Data are represented in mean ± S.D. and were analyzed using the one-way ANOVA test. (DOCX 278 kb) Additional file 2: Raw data of MTT assays. (XLSX 10 kb) Additional file 3: Raw data of cell proliferation using flow cytometry. (XLSX 14 kb) Additional file 4: Raw data of gene expression by real time RTPCR. (XLSX 17 kb)

## Abbreviations

A.S.: *Allium sativum L.*
ARNm: ARN messenger
CFSE: 5, 6-carboxyfluorescein diacetate succinimidyl ester
Con A: Concanavalin A
Ct: Threshold cycles
IL: Interleukin (e.g., IL-4, IL-17)
mAb: Monoclonal antibody
MTT: 3-(4, 5 dimethylthiazol-2-yl)-2, 5 diphenyltetrazolium bromide
PBMCs: Peripheral blood mononuclear cells
PHA: Phytohaemagglutinin
Th: T helper
