# Supplementary material for: Allium sativum L. regulates in vitro IL-17 gene expression in human peripheral blood mononuclear cells
Source: BMC Complement Altern Med. 2016 Sep 29;16:377. doi: 10.1186/s12906-016-1365-9 (PMC5041567; doi:10.1186/s12906-016-1365-9)

Supplementary figure 1:

a b

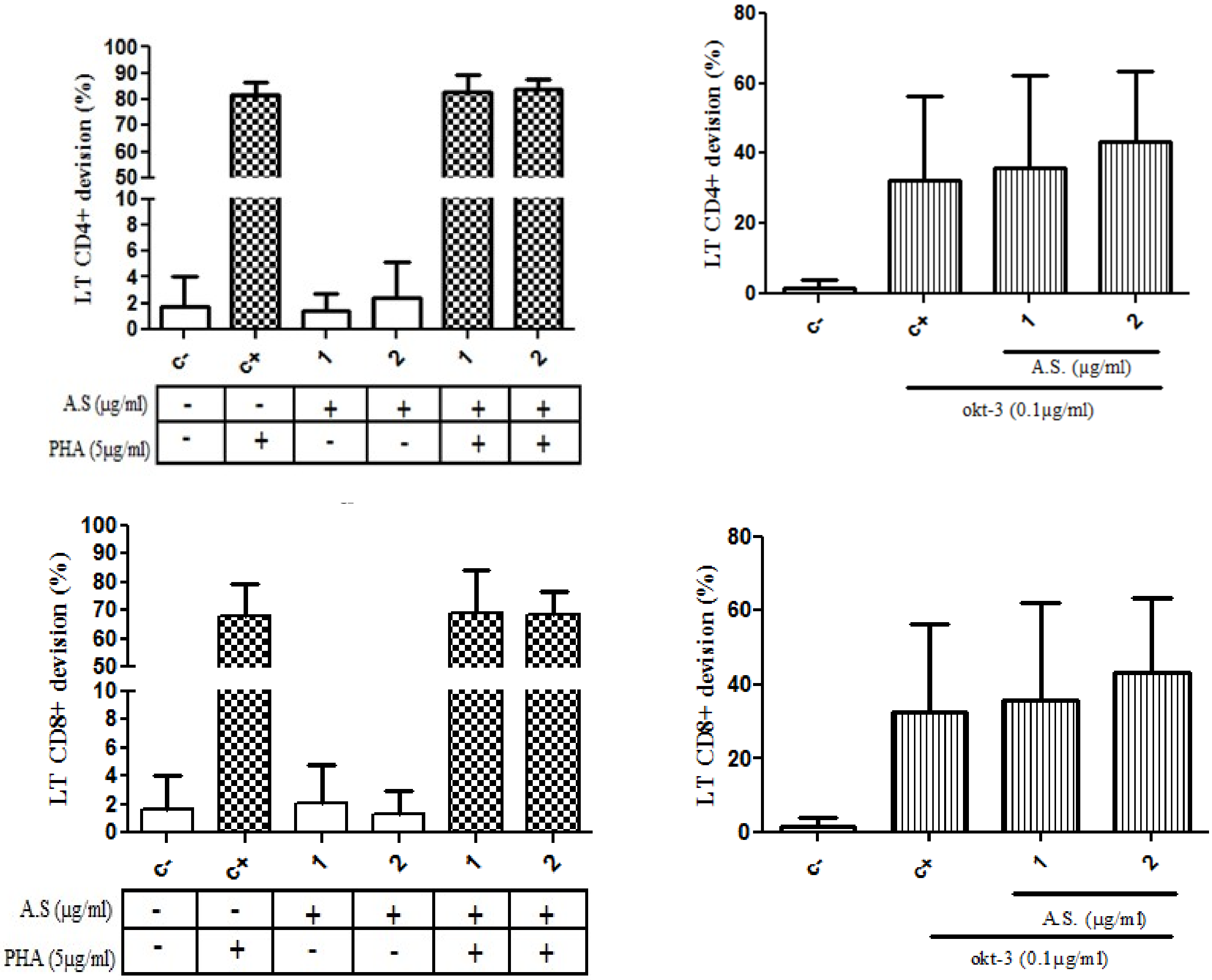

Supplement: Supplementary file 1 [file 12906_2016_1365_MOESM1_ESM.docx]
